# Adaptation to Bleaching: Are Thermotolerant Symbiodiniaceae Strains More Successful Than Other Strains Under Elevated Temperatures in a Model Symbiotic Cnidarian?

**DOI:** 10.3389/fmicb.2020.00822

**Published:** 2020-05-05

**Authors:** Casandra R. Newkirk, Thomas K. Frazer, Mark Q. Martindale, Christine E. Schnitzler

**Affiliations:** ^1^Whitney Laboratory for Marine Bioscience, University of Florida, St. Augustine, FL, United States; ^2^Fisheries and Aquatic Sciences Program, School of Forest Resources and Conservation, University of Florida, Gainesville, FL, United States; ^3^School of Natural Resources and Environment, University of Florida, Gainesville, FL, United States

**Keywords:** Jellyfish, strobilation, symbiosis, thermotolerance, *Cassiopea*, holobiont, warming

## Abstract

The ability of some symbiotic cnidarians to resist and better withstand stress factors that cause bleaching is a trait that is receiving increased attention. The adaptive bleaching hypothesis postulates that cnidarians that can form a stable symbiosis with thermotolerant Symbiodiniaceae strains may cope better with increasing seawater temperatures. We used polyps of the scyphozoan, *Cassiopea xamachana*, as a model system to test symbiosis success under heat stress. We sought to determine: (1) if aposymbiotic *C. xamachana* polyps could establish and maintain a symbiosis with both native and non-native strains of Symbiodiniaceae that all exhibit different tolerances to heat, (2) whether polyps with these newly acquired Symbiodiniaceae strains would strobilate (produce ephyra), and (3) if thermally tolerant Symbiodiniaceae strains that established and maintained a symbiosis exhibited greater success in response to heat stress (even if they are not naturally occurring in *Cassiopea*). Following recolonization of aposymbiotic *C. xamachana* polyps with different strains, we found that: (1) strains *Smic*, *Stri, Slin*, *and Spil* all established a stable symbiosis that promoted strobilation and (2) strains *Bmin1* and *Bmin2* did not establish a stable symbiosis and strobilation did not occur. Strains *Smic*, *Stri*, *Slin*, and *Spil* were used in a subsequent bleaching experiment; each of the strains was introduced to a subset of aposymbiotic polyps and once polyp tissues were saturated with symbionts they were subjected to elevated temperatures - 32°C and 34°C - for 2 weeks. Our findings indicate that, in general, pairings of polyps with Symbiodiniaceae strains that are native to *Cassiopea (Stri* and *Smic*) performed better than a non-native strain (*Slin*) even though this strain has a high thermotolerance. This suggests a degree of partner specificity that may limit the adaptive potential of certain cnidarians to increased ocean warming. We also observed that the free-living, non-native thermotolerant strain *Spil* was relatively successful in resisting bleaching during experimental trials. This suggests that free-living Symbiodiniaceae may provide a supply of potentially “new” thermotolerant strains to cnidarians following a bleaching event.

## Introduction

Endosymbiosis between dinoflagellate algae in the family Symbiodiniaceae and cnidarian hosts is common among many members of the phylum Cnidaria (Muscatine and Porter, 1977; Davy et al., 2012). Symbiodiniaceae cells harbored in the endodermal tissues of cnidarians provide their hosts with nutritional compounds via photosynthesis and, in turn, they are provided protection from potential consumers and access to compounds essential to the photosynthetic process (Muscatine, 1990; Gattuso et al., 1999; Burriesci et al., 2012). The symbiosis between cnidarians and these dinoflagellate algae has been characterized mainly in stony corals due, in large part, to the fact that coral reefs are highly vulnerable to increased ocean warming associated with a changing climate (Walther et al., 2002; Douglas, 2003; Hughes et al., 2003) and thus is a focus of concern and research. Vulnerability and death of symbiotic coral hosts, is exacerbated by the expulsion of symbionts from host tissues. This expulsion and loss of symbionts is known as “bleaching” and is one of the most pressing environmental issues facing contemporary marine resource managers (Hoegh-Guldberg, 1999; West and Salm, 2003; Ainsworth et al., 2016; Genevier et al., 2019). Although bleaching is a widespread cause of death of symbiotic corals, some hosts seem to be able to better withstand stress factors that typically lead to a bleaching event (Mumby and Steneck, 2011). The “adaptive bleaching hypothesis,” a hypothesis formulated around these survivors, postulates that some cnidarian hosts have the capacity to establish and maintain a stable symbiosis with strains of heat tolerant Symbiodiniaceae following a bleaching event and, in doing so, are likely to exhibit increased resistance to future bleaching events (Kinzie et al., 2001; Fautin and Buddemeier, 2004; Yamashita et al., 2014). In some cases, researchers have observed characteristics of the host which may contribute to resistance to increasing temperatures which include: growth form of the host, physiology of the host (such as producing fluorescent pigments that absorb and scatter solar radiation reducing the severity of bleaching), and changes in gene expression at prolonged high temperatures that allow hosts to better withstand high temperatures (Baird and Marshall, 2002; McClanahan et al., 2004; Baird et al., 2009; Palumbi et al., 2014). Typically, however, characteristics of the algae are attributed with primary importance (Loya et al., 2001; Bhagooli and Hidaka, 2003; Stat and Gates, 2011; Hume et al., 2015; Cziesielski et al., 2018).

Previous reports that some coral colonies were less susceptible to heat stress and recovered more rapidly than sympatric taxa, led to a determination that certain strains of Symbiodiniaceae are more tolerant to heat stress than others (Berkelmans and van Oppen, 2006; LaJeunesse et al., 2009, 2018; Pettay et al., 2015). Currently, there are nine distinct genera of the family Symbiodiniaceae that have been identified and shown to form relationships with different species of invertebrates; these genera are each compromised of multiple species (or strains) (LaJeunesse et al., 2018). Studies focused on the morphology and physiology of Symbiodiniaceae have determined that each genus (and even particular species within a genus) has distinct characteristics that can include traits such as tolerance to high temperatures or light levels (Sampayo et al., 2008; Díaz-Almeyda et al., 2017; Grégoire et al., 2017; Lesser et al., 2019). For example, certain strains of *Durusdinium* have shown higher thermotolerance and have been found in coral hosts occupying outlying habitats where environmental conditions are less favorable, and also in corals recovering from previous bleaching events (Thornhill et al., 2006b; Poquita-Du et al., 2020). Although certain genera can have a general tolerance scale to heat (such as seen in *Durusdinium*), other factors play a role in the heat tolerance of a strain with one major influence being the host that a strain associates with (Hoadley et al., 2019). Studies have shown that strains that have been deemed less tolerant to heat (such as members of the genera *Cladocopium*) (Baker, 2004) can perform better at high temperatures when paired with a particular host, and allow the host to be less susceptible to bleaching (Hume et al., 2013; Ziegler et al., 2017; Howe-Kerr et al., 2019).

Focusing on the ability of certain Symbiodiniaceae strains to resist photosynthetic breakdown at high temperatures has led many researchers to design studies to test the ability of a cnidarian host to successfully form and maintain a stable symbiosis with particular types of symbiont species. In general, most cnidarian hosts display partner specificity during the adult stage for one of the nine Symbiodiniaceae genera or for particular species or even strains within a genus (Coffroth et al., 2001, 2010; Weis et al., 2001; LaJeunesse et al., 2003; Stat et al., 2009; Putnam et al., 2012). For example, the model cnidarian *Cassiopea xamachana*, or the upside-down jellyfish, is most commonly found in symbiosis with *Symbiodinium microadriaticum* (cp-23S type A194; ITS2 type A1). The culture CassKB8 is one clonal strain of this symbiont type that was used in our experiments (Thornhill et al., 2006a; Mellas et al., 2014). *Cassiopea xamachana* has a life cycle that includes both a juvenile polyp stage (in which the mouth is formed and asexual budding occurs) and an adult medusa stage (Hofmann and Gottlieb, 1991; Hofmann et al., 1996; Fitt and Costley, 1998). Although adult *C. xamachana* are most commonly found to harbor this single strain, experimental studies have shown that during the polyp stage, hosts are flexible in the strain of symbiotic algae they acquire (Fitt, 1985; Mellas et al., 2014). Previous studies have determined that the flexibility of *Cassiopea* polyps allows them to obtain strains from multiple Symbiodiniaceae genera (*Symbiodinium, Breviolum, Cladocopium*, and *Durusdinium)*, maintain a symbiosis with these strains, and metamorphose into ephyra (Thornhill et al., 2006a; Mellas et al., 2014). This flexibility of *C. xamachana* polyps, along with the ease of maintaining them in a laboratory setting, makes them ideal candidates to test the establishment and maintenance of symbiosis with different Symbiodiniaceae strains and their performance under heat stress.

Other characteristics of *C. xamachana* further add to its appeal as a model system (Ohdera et al., 2018). One advantage is that a developmental change occurs when symbiotic stability has been reached in *C. xamachana* (Colley and Trench, 1983; Fitt, 1985). During the polyp stage of development, hosts must acquire algal cells from the environment to grow and transition into the next stage of their life cycle. This life cycle change, known as strobilation, is entirely dependent on a stable symbiosis, and is a precursor to the ephyra stage (an immature medusa) (Hofmann et al., 1996; Fitt and Costley, 1998). This developmental change from a polyp to an ephyra following a suitable interaction with certain Symbiodiniaceae strains can tell us if the partnership is stable enough (at least in the short term to provide the necessary trigger for strobilation) (Hofmann et al., 1996). Introduction of thermotolerant symbionts to bleached cnidarian hosts may be a plausible step in helping stressed cnidarians recover from a bleaching event, even if the symbiosis is only maintained in the short term (Lewis and Coffroth, 2004; Silverstein et al., 2015). If the partners are not working together, i.e., if the symbionts are not maintained or do not saturate host tissues, then the thermotolerance of the algal strain will be inconsequential in the long term (Goulet, 2006).

Because of the characteristics of the symbiosis between Symbiodiniaceae and *C. xamachana* polyps outlined above, we chose to use this system to study the success of various symbiont strains in polyps at optimal (25°C) and elevated temperatures (32 and 34°C). We used aposymbiotic polyps to investigate the ability of symbiont-free polyps to acquire different strains of Symbiodiniaceae and strobilate. Strains used for both the acquisition experiments and experiments at elevated temperatures were strains native to *Cassiopea spp.* (homologous) and non-native strains (heterologous). These Symbiodiniaceae strains also ranged from heat sensitive to more heat tolerant to previous studies. After determining which strains maintained a symbiosis with polyps and promoted strobilation, we tested bleaching susceptibility of the different host-symbiont combinations under high temperature stress. Though the general thermotolerance of these strains has been documented (Suggett et al., 2008; Díaz-Almeyda et al., 2017), the tolerance of Symbiodiniaceae strains to heat likely changes depending on which cnidarian host they are partnered with (Goulet, 2006, 2015; Mieog et al., 2009; Hoadley et al., 2019). Accordingly, we hypothesized there were two possible outcomes for our experiments. The first outcome could be that native symbiont strains from *Cassiopea spp.* perform better under high temperatures when compared to a non-native strain (regardless of whether or not a non-native strain has shown a higher tolerance to heat previously) because they have evolved a partner specificity. Partner specificity has been documented in many other cnidarian systems and is one of the main limitations to symbiont reshuffling (Gabay et al., 2019; Medrano et al., 2019). Alternatively, because *C. xamachana* polyps are documented to be more flexible when acquiring symbiont strains, the second outcome could be that a non-native strain with increased thermal tolerance (Table 1) performs better in maintaining a stable symbiosis and possibly provides polyps with a greater resistance and/or resilience to future bleaching events. This second scenario is plausible as many cnidarian species have shown the ability to be flexible in the type of symbiont strain that they take in as juveniles (Cumbo et al., 2013; Cunning et al., 2013; Mellas et al., 2014; Ng et al., 2019).

**TABLE 1:** Cultures used in experimental inoculations of aposymbiotic *C. xamachana* polyps: Genus and species name, species code, culture ID, cnidarian host that culture was isolated from, strain name, and general tolerance to high temperatures.

**Symbiodiniaceae type**	**Species Code**	**Culture**	**Host isolated from**	**Location**	**ITS2 Type**	**Heat tolerance**
*Symbiodinium microadriaticum*	*Smic*	CassKB8	*Cassiopea xamachana*	North Pacific	A1	High (Díaz-Almeyda et al., 2017)
*Symbiodinium tridacnidorum*	*Stri*	CassEL1	*Cassiopea spp.*	North Pacific	A3	Low (Díaz-Almeyda et al., 2017)
*Breviolum minutum*	*Bmin1*	rt-002	*Aiptasia pallida*	Caribbean	B1	Low (Suggett et al., 2008)
*Breviolum minutum*	*Bmin2*	Mf 1.05b	*Orbicella faveolata*	Caribbean	B1	Low (Suggett et al., 2008)
*Symbiodinium linuchae*	*Slin*	rt-379	*Aiptasia pallida*	Caribbean	A4	High (Díaz-Almeyda et al., 2017)
*Symbiodinium pilosum*	*Spil*	Zs	No host- free living strain	Caribbean	A2	High (Díaz-Almeyda et al., 2017)

*Heat tolerance was obtained in these studies from algal cells in culture.*

## Materials and Methods

### Collection and Initial Bleaching of *Cassiopea xamachana* Polyps

We obtained *C. xamachana* adult medusa from the waters surrounding the Keys Marine Laboratory in Layton, Florida (24.8263° N, 80.8139° W) and housed them for the duration of the study at the Whitney Laboratory for Marine Bioscience (St. Augustine, FL, United States). We kept medusa at 25°C on a 13:11 light cycle in aquaria with flow through seawater, with feedings of *Artemia* occurring three times weekly. Larvae from brooding medusa were allowed to settle on the walls of aquaria where they transitioned into polyps. We collected a subset of these polyps for subsequent laboratory experiments. Collected polyps were placed in 48-well plates, each well contained 1 mL of 0.2-μm filtered seawater and one individual per well. 48-well plates were kept at 35.5°C in complete darkness for 2–3 weeks to generate aposymbiotic individuals (Newkirk et al., 2018). We verified that polyps were completely symbiont free via confocal microscopy at 647 nm (autofluorescence of any residual algae can be seen at this wavelength). We continued the heat treatment for an additional week when necessary. Polyps that were determined to be completely symbiont free were placed into new 48-well plates. As before, each well contained 1 mL of 0.2-μm filtered seawater and one polyp. 48-well plates were maintained at 25°C in complete darkness for one week until the animals were needed for recolonization experiments. Symbiont-free polyps were fed *Artemia* three times weekly and checked periodically to ensure that they remained in a symbiont-free state.

### Inoculation of *Cassiopea xamachana* Polyps With Symbiodiniaceae Strains

**FIGURE 1:**
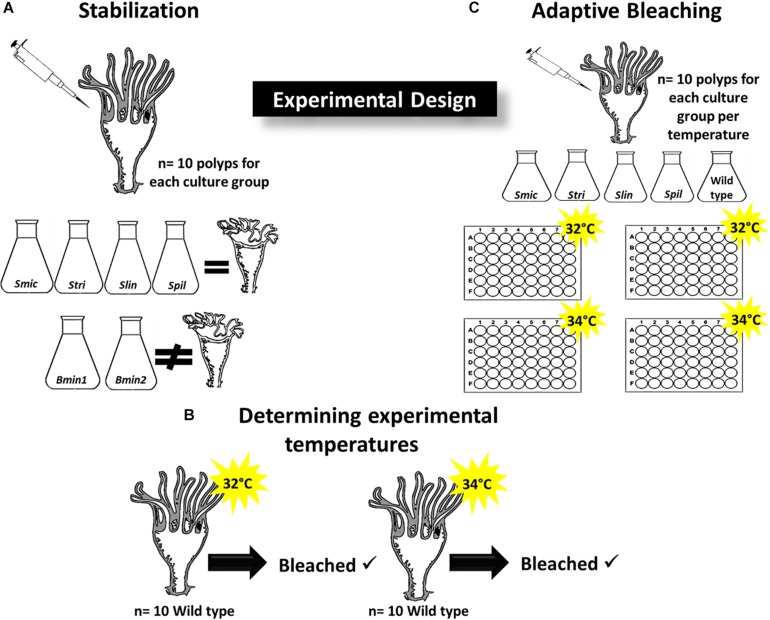
Experimental Design. **(A)** Determining Symbiodiniaceae strains that induce strobilation in inoculated polyps (*n* = 10 polyps per group); culture strains *Smic*, *Stri*, *Slin, Spil* all induced strobilation while *Bmin1* and *Bmin2* did not maintain symbiosis and no strobilation occurred. **(B)** Wild type polyps were placed at 32 and 34°C (*n* = 10 per temperature) to determine if bleaching of symbionts occurred; each temperature induced bleaching, and these were used for adaptive bleaching trials. **(C)** Culture strains that induced bleaching used for adaptive bleaching experiments at 32 and 34°C (*n* = 10 polyps per temperature group); two 48-well plates per temperature group.

In *C. xamachana* polyps, strobilation is an indication of a successful symbiosis establishment (Colley and Trench, 1983), so the ability of introduced strains to remain in host tissues was established before polyps could be placed again at experimental bleaching temperatures. We tested six algal strains, with each strain known to exhibit different tolerances to high temperatures (Table 1). The six strains tested *were*: (1) *Symbiodinium microadriaticum* (ITS2 type A1), (2) *S. tridacnidorum* (ITS2 type A3), (3) *S. linucheae* (ITS2 type A4), (4) *S. pilosum* (ITS2 type A2), (5) *Breviolum minutum* 1 (ITS2 type B1), and (6) *B. minutum* 2 (ITS2 type B1). *Symbiodinium microadriaticum* (*Smic), S. tridacnidorum* (*Stri*), *S. pilosum* (*Spil*), *B. minutum* 2 (*Bmin2*) strains were isolated and described by R.A. Kinzie III and M.A. Coffroth (Santos et al., 2002; Voolstra et al., 2009; Bayer et al., 2012). Strain *B. minutum* 1 (*Bmin1*) was isolated originally by D.A. Schoenberg and described in detail in LaJeunesse et al. (2012). Lastly, *S. linucheae* (*Slin*) was described originally in Trench and Thinh (1995). We obtained strains *Smic*, *Stri*, and *Bmin2* from the University of Buffalo BURR culture collection; *Slin and Spil* were obtained from Virginia Weis (Oregon State University) and *strain Bmin1* was obtained from Monica Medina (Penn State University). All cultures received were genotyped prior to shipment at the ITS2 region to confirm species membership. We grew and maintained Symbiodiniaceae cultures at 25°C on a 13:11 light cycle in F/2 medium (Guillard and Ryther, 1962). Symbiont-free polyps were separated into six groups (*n* = 10 in each group) and kept in separate 48-well plates, each well contained 1 mL of 0.2-μm filtered seawater and one polyp. To establish large numbers of each culture strain in host tissues, we inoculated polyps in each group with ∼2000 cells ml^–1^ of culture, concurrent with feeding of *Artemia* to allow polyp mouths to open; we performed these inoculations with feedings three times a week. After each 24 h inoculation, we rinsed all polyps and placed them in fresh 48-well plates which were maintained at 25°C on a 13:11 light cycle. Inoculations continued for 6 weeks (three times weekly), after which time we stopped inoculations and monitored polyps to determine if symbionts would remain inside host tissues (and lead to strobilation- seen after approximately 3–4 weeks for each strain) or if symbiont numbers would decrease and the symbiosis would be compromised (Figure 1A). Establishment of symbiosis was determined via confocal microscopy.

### Determination of Experimental Bleaching Temperatures

Prior to reinoculation of symbiont-free polyps with strains of Symbiodiniaceae for bleaching trials, we assessed if polyps would bleach at temperatures commonly used in laboratory adaptive bleaching experiments. The selected temperatures were 32 and 34°C, as both temperatures are known to be above the threshold at which bleaching is induced (Gates et al., 1992; Robinson and Warner, 2006; McGill and Pomory, 2008). We collected twenty wild type polyps (polyps that are native and non-bleached) from aquarium tanks for this purpose (*n* = 10 polyps exposed to both temperatures). Before bleaching, we imaged each of the polyps at all focal planes using the z-stack feature on a confocal microscope; autofluorescence of Symbiodiniaceae in host tissues was captured at 647 nm. We employed the “spots” function of Imaris software (Bitplane, Inc.) to determine the number of symbionts housed in host tissues using the uploaded z-stack image (Newkirk et al., 2018). Once we established symbiont numbers in each polyp at the ambient temperature of 25°C, we placed the polyps in 48-well plates (one polyp per well) and placed the plates in incubators directly at the designated temperatures (one group at 32°C and one group at 34°C). After 10 days, we removed polyps and imaged them again to count remaining symbionts as described above. Polyps in each group showed significant decreases in symbiont number, so these temperatures were determined to be adequate for subsequent bleaching experiments (Figure 1B).

### Reinoculation of Symbiont-Free Polyps and Adaptive Bleaching Trials

To assess the effect of different algal strains on the ability of *C. xamachana* polyps to resist bleaching from future exposure to high temperatures, we inoculated aposymbiotic polyps with the strains *Smic*, *Stri*, *Slin*, and *Spil*.

For adaptive bleaching trials, we used 20 symbiont-free polyps for each treatment group, and kept polyps for each group in separate 48-well plates (four experimental inoculation plates in total); each well contained 1 mL of 0.2-μm filtered seawater and one polyp. We performed inoculations for each treatment group separately to ensure no cross contamination occurred. We inoculated polyps with algal cells as described above. Polyps were fed three times weekly over the course of 5 weeks to allow the density of symbionts to increase quickly inside host tissues. We selected only polyps harboring at least > 1000 algal cells at the end of inoculation periods to use for adaptive bleaching trials. Polyps from each algal culture group were divided into two smaller groups, each with 10 polyps, for use in experiment trials at 32 and 34°C. Before exposing the polyps to high temperature, we imaged polyps from each group (once again using confocal microscopy at 647 nm) and counted the exact numbers of symbionts using Imaris software. Following these initial counts, we placed polyps in 48-well plates using a random assignment generator. Polyps from each inoculation group, along with wild type polyps, were distributed between the two plates at each temperature such that each plate contained 5 polyps from each group. We then subjected the plates with polyps to the experimental temperatures; plates with polyps were maintained in the dark without allowing polyps access to food (Figure 1C). Over the course of 2 weeks at each temperature, we removed individual polyps from wells at three separate time points (3, 9, and 14 days after start of each trial) to image and count symbionts. After the 2-week bleaching period, polyps were returned to ambient conditions. We used the recorded number of symbionts counted at each time point to calculate the percentage of symbionts lost from each polyp over the course of our experiment.

### Statistical Analysis

Data were normalized and transformed using a natural log transformation. Data were analyzed using a mixed model ANOVA approach (lme4 package, Bates et al., 2014). Loss of algal cells was the dependent variable (symbiont counts during experiment), while our within-subjects factor was “time” and our between-subjects factor was “inoculation group” (i.e., groups *Stri*, *Smic*, *Slin*, and *Spil*). We then employed pairwise Tukey’s *post hoc* tests. In all cases, the level of statistical significance was set at *p* = 0.05. All statistical analyses were carried out using the RStudio software package (Version 1.2.1335-1, R Core Team, 2019).

## Results

### Inoculation of Bleached Polyps and Establishment of a Stable Symbiosis

**FIGURE 2:**
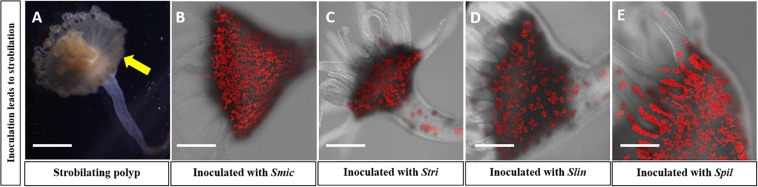
Confocal microscopy confirming high concentration of symbionts in four reinoculated polyp groups; autofluorescence of (red) algal cells seen. Strobilation of polyps was seen in these groups: **(A)** Polyp currently strobilating; yellow arrow displays ephyra pinching off from polyp. **(B)** Polyp reinoculated with *Smic* culture. **(C)** Polyps reinoculated with *Stri* culture. **(D)** Polyps reinoculated with *Slin* culture. **(E)** Polyps reinoculated with *Spil* culture (Scale bar = 0.5 mm).

**FIGURE 3:**
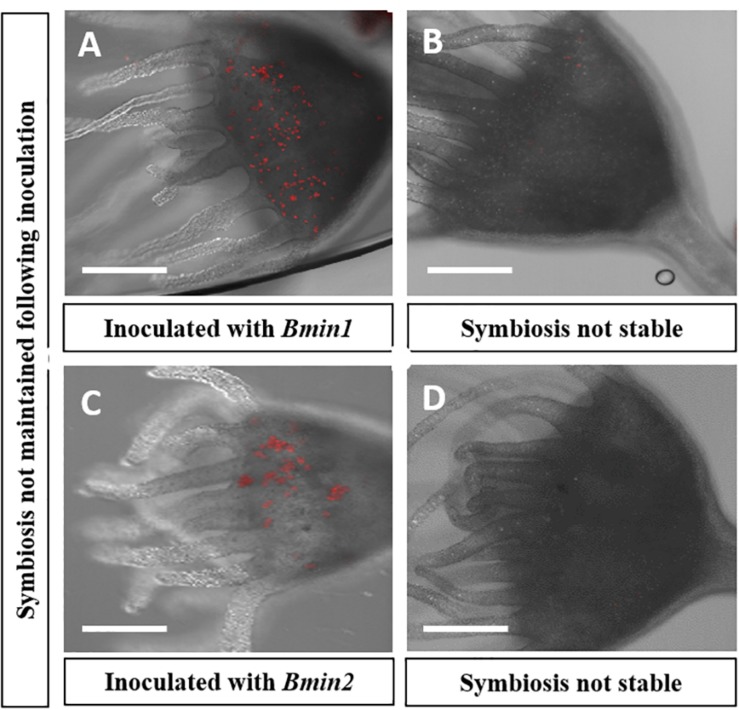
Confocal microscopy displaying loss of algal cells following inoculations in two culture groups; autofluorescence of (red) algal cells seen: **(A)** Polyp inoculated with *Bmin1* culture. **(B)** Loss of symbiosis in *Bmin1* inoculated polyp. **(C)** Polyps inoculated with *Bmin2* culture. **(D)** Loss of symbiosis in *Bmin2* inoculated polyp (Scale bar = 0.5 mm).

All four *Symbiodinium* species strains of algae, i.e., *Smic*, *Stri*, *Slin*, and *Spil*, were able to establish a stable symbiosis with their host polyps (Figure 2A). Strobilation occurred within 5–6 weeks for polyps reinoculated with *Smic*, *Stri*, *and Slin* (Figures 2B–D). Polyps inoculated with *Spil* (a free-living strain of) strobilated between 11 and 12 weeks (Figure 2E). We also observed asexual budding in all polyps inoculated with these strains. Polyps inoculated with algal strains *Bmin1* and *Bmin2* were not able to maintain a stable symbiosis. Polyps representing these latter two groups were observed to take in cells during feeding events, but the algal cells were ultimately rejected by the host tissues (Figure 3). Thus, these two *Breviolum* Symbiodiniaceae strains were not used in subsequent experiments.

### Adaptive Bleaching Trials

All experimental groups, i.e., *Smic*, *Stri*, *Slin*, *Spil*, and wild type, exhibited significant loss of algal cells by the end of the 2-week bleaching period regardless of temperature. Although substantial loss of algal symbionts was documented (15% of loss of algal cells on average comes from starvation and being held in the dark), notable differences among treatment groups at different time points were recorded over the course of the 2-week experiment.

#### Comparison of Loss of Symbiodiniaceae Strains at 32°C

**FIGURE 4:**
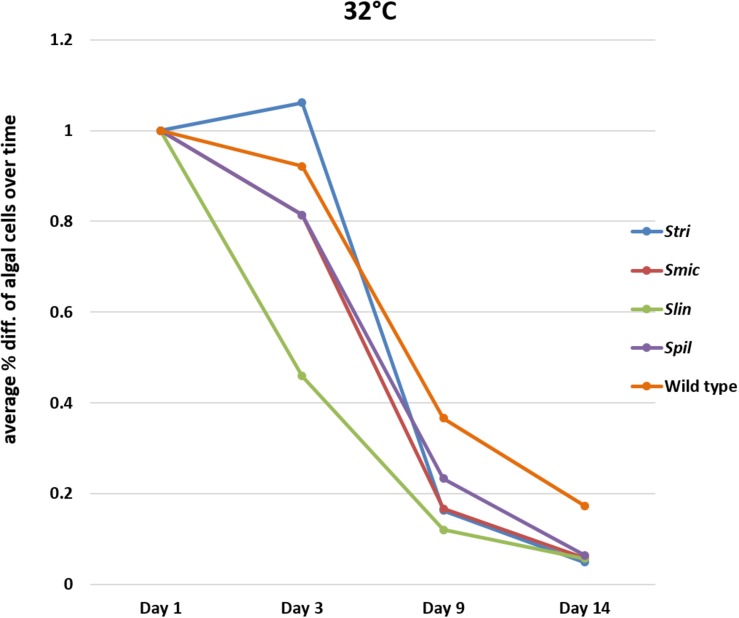
Average percent loss of algal cells at each time point for each algal strain at 32°C (SD error bars omitted to allow visual clarity).

In bleaching trials conducted at 34°C, the Cassiopea native strain *Stri* lost, on a percentage basis, fewer algal cells on average compared to initial counts of cells by day three at temperature. A similar percentage was seen when comparing loss of these strains to one of the non-native strains used in our trials; *Spil* (a traditionally free-living strain) lost on average 23% of algal cells by day three relative to initial algal cell counts. The third native strain *Smic*, along with non-native strain *Slin*, saw a greater percentage of algal cells being lost on average with 31 and 54% of cells lost, respectively, by day three. By day nine, two *Cassiopea* native strains (*Smic* and *Stri*) were observed to lose 83% of algal cells on average relative to initial counts. In contrast, the *Cassiopea* native wild type strain retained a higher number of algal cells losing only 66% of cells on average. The remaining non-native strains again displayed different performance values with *Spil* polyps losing 77% of algal cells on average (outperformed only by wild type cells), and the *Slin* polyps losing 88% of algal cells on average (maintaining the trend of greatest loss overall). By our final time point at day 14, the major trend observed for all strains was almost complete expulsion of remaining algal cells. Strains *Smic, Stri, Spil*, and *Slin* all lost on average 94% of algal cells relative to initial counts. In contrast to all other groups, wild type polyps at this time point lost only 83% of algal cells relative to initial symbiont numbers (Figure 4 and Supplementary Tables S1–S5).

#### Comparison of Loss of Symbiodiniaceae Strains at 34°C

**FIGURE 5:**
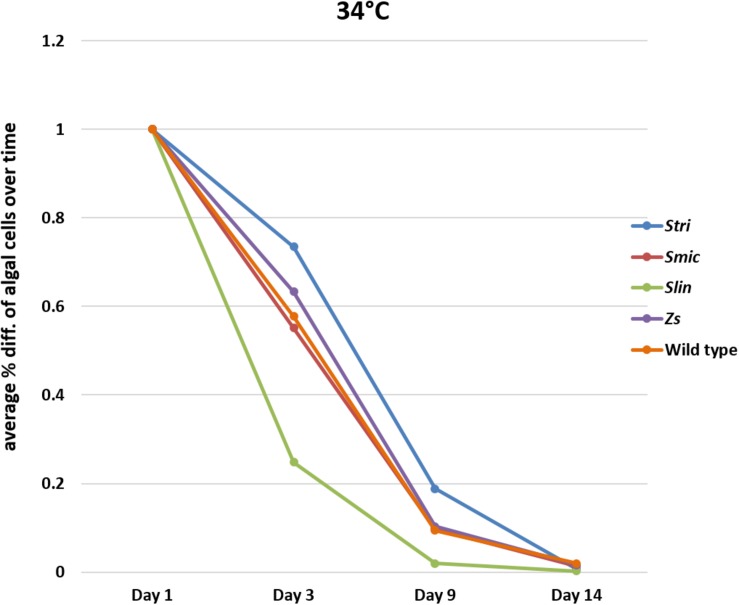
Average percent loss of algal cells at each time point for each algal strain at 34°C (SD error bars omitted to allow visual clarity).

In bleaching trials conducted at 34°C, the *Cassiopea* native strain *Stri* lost, on a percentage basis, fewer algal cells on average than the other native strains in our trials with 26% of algal cells expelled by day three. The native strains, *Smic* and wild type, performed similarly to one another with 44% of algal cells expelled by day three. As seen at 32°C, polyps inoculated with the free-living *Spil* outperformed some of the native strains, losing only 37% of algal cells on average by day three. In contrast, the non-native strain *Slin* remained the strain with the highest number of cells expelled at day three, with 75% of algal cells expelled relative to initial counts. The percent algal cell loss at days nine and 14 were similar for each Symbiodiniaceae strain. On day 9, all strains had lost on average 91% of algal cells relative to starting counts and by day 14 each strain saw 99% of algal cells expelled relative to starting counts (Figure 5 and Supplementary Tables S6–S10).

#### Statistical Comparisons of Inoculated Groups Following Adaptive Bleaching Trials

At 32°C, *Slin* was outperformed by all other strains, though the rate of cell loss among the strains was not deemed to be statistically significant (ANOVA, *p* > 0.05; see Supplementary Table S11 for pairwise comparisons). At 34°C, strain *Slin* was significantly outperformed by *Stri*, *Smic*, and *Spil*, but not significantly by wild type cells (ANOVA, *p* < 0.05; see Supplementary Table S11 for pairwise comparisons). We also found that survivorship, in general, was statistically higher for polyps held at 32°C bleaching conditions relative to those bleached at 34°C (ANOVA, *p* < 0.05).

## Discussion

### Inoculation of Symbiont-Free Polyps and Establishment of a Stable Symbiosis

The formation of a symbiotic relationship between a cnidarian host and algal cells requires numerous stages, including initial contact between potential partners, acquisition of algal cells by the host, and most importantly, stability and division of algal cells within host tissues (Davy et al., 2012). In *C. xamachana*, stability of the symbiotic relationship between partners is marked by a metamorphic change from polyp to ephyra (Hofmann et al., 1996; Fitt and Costley, 1998). Another marker that can be seen in *C. xamachana* polyps is asexual budding in accordance with strobilation. Asexual budding alongside continuation of the life cycle when inoculated with a Symbiodiniaceae strain is a strong measure of the health of the symbiotic relationship. Although it had been determined previously that *C. xamachana* polyps are flexible in the initial uptake of algal cells (Mellas et al., 2014), the results presented in this study clearly indicated that symbionts initially acquired were not necessarily maintained over time. Our inoculation experiments, which included six different Symbiodiniaceae strains (*Smic, Stri*, *Slin*, *Spil*, *Bmin1*, and *Bmin2*), yielded only four successful pairings. Non-native *strains Bmin1 and Bmin2* were ultimately rejected by their host polyps. Both of the rejected algal cell types were *B. minutum* strains and showed the same pattern of initial uptake of many algal cells before being expelled from host tissues. This may have been due to an inability to overcome the innate immunity of the host or the lack of algal proliferation (Davy et al., 2012; Wolfowicz et al., 2016). Additional factors that might have contributed to the inability of these *B. minutum* strains to colonize polyps is both algal cell size and the overall metabolic benefits of the cells to the host (Biquand et al., 2017; Gabay et al., 2018). Biquand et al. (2017) found that Symbiodiniaceae strains that are larger in size were less likely to colonize *Aiptasia* hosts, and size of an algal strain plays a role in the overall selectivity by a cnidarian host. In Starzak et al. (2014) it was observed that heterologous symbiont cells in *Aiptasia* can increase respiration in the host and this increased cost can outweigh any benefits provided by the algal strain. While we did not determine the specific reason why *B. minutum* strains did not colonize *C. xamachana* polyps, future experiments could possibly reveal selective mechanisms as outlined in these studies.

The inability of two *B. minutum* strains to remain inside host tissues is important in relation to the goals of our study. Testing the adaptive capability of a Symbiodiniaceae strain requires first understanding if the algal strain of interest will remain in symbiosis with its cnidarian host. For example, many strains of *Durusdinium* are known to have a higher temperature tolerance than other Symbiodiniaceae (Berkelmans and van Oppen, 2006; Thornhill et al., 2006b). The thermotolerance of this genus makes it very appealing to scientists aiming to promote or facilitate bleaching resistance of symbiotic cnidarians through use of better adapted Symbiodiniaceae (Stat and Gates, 2011; Silverstein et al., 2017). If algal cells from this genus do not maintain a stable symbiosis with the cnidarian hosts at risk, however, then the thermotolerance will be of little consequence.

Of interest was our finding that a strain not typically known to form a symbiosis with cnidarians was able to establish a stable symbiosis with *C. xamachana* host polyps, as evidenced by strobilation. Strain *Spil* was originally isolated from the surface of *Zoanthus sociatus*, and is considered a non-symbiotic species (Trench and Blank, 1987). Our observations that a thermotolerant strain that has been deemed to be generally non-symbiotic and usually found in a “free-living” state, was able to not only establish itself within *C. xamachana* polyps, but trigger strobilation suggests that these algal cells that are known to spend the majority of their life history in a free-living state might serve as a reservoir of Symbiodiniaceae available to assist bleached cnidarians in their recovery. The abundance of these free-living cells can be altered however (in terms of strains found in an environment), so the availability of certain temperature tolerant strains that can be acquired by hosts must be taken into account (Cumbo et al., 2018). When Symbiodiniaceae cells are found in a free-living state, they typically reside in the benthos near or around reef communities (Littman et al., 2008), and an abundant diversity of types of free-living Symbiodiniaceae in the sediment (and to a lesser degree in the water column) has been documented (Manning and Gates, 2008; Granados-Cifuentes et al., 2015). In a study performed on Symbiodiniaceae cultures by Nitschke et al. (2015), researchers determined that there was a significant difference in response to temperature between a non-symbiotic *Symbiodinium* strain (found free-living in the sediment) and a *Symbiodinium* strain that is usually found in symbiosis with a cnidarian host. The non-symbiotic strain was less impacted by high temperature and remained in a fairly stable growth phase during experimental trials due to the selection pressures of living free in the environment, while the endosymbiotic strain begin to break down much faster at high temperatures. This finding is consistent with results presented here. Although typically free-living strain *Spil* ultimately did not persist under the extreme experimental conditions (high temperature, no light, and no supplemental feeding of polyps for a 2-week period), it did remain in higher numbers in host tissues at both temperatures in comparison to symbiotic culture strains used (strain *Slin*). This is again likely due to lifestyle differences between strains living freely and cultured strains that can give rise to differences in thermotolerance (Nitschke et al., 2015). Previous studies have shown that strains that typically are free-living maintain the ability to establish a symbiosis with cnidarian hosts that are greatly impacted by high temperatures, notably *Acropora spp.* (Coffroth et al., 2006; Adams et al., 2009; Cumbo et al., 2013). These studies demonstrated the ability of *Acropora* larvae to acquire Symbiodiniaceae from the sediment, showing that these strains could provide a source of algal cells following bleaching and lead to recovery of coral populations. Because we have provided evidence in our study that acquisition of new algal partners following bleaching can include Symbiodiniaceae that are generally free-living (or non-symbiotic), this potentially better adapted stock of Symbiodiniaceae cells must be considered when discussing increasing the adaptive ability of symbiotic cnidarians. These algal cells possibly retain the potential, if nothing else, to provide bleached cnidarians with a temporary reservoir of symbionts that promote short-term recovery until conditions stabilize or the host has the chance to reacquire a high number of “native” symbionts.

### Adaptive Bleaching Trials

Increasing resistance and/or resilience to high temperature events is one of the many mechanisms proposed to help symbiotic cnidarians (in most cases symbiotic hermatypic corals) recover following a bleaching event (West and Salm, 2003; Rosado et al., 2019). One of the main components thought to be involved in increasing the resilience of symbiotic cnidarian hosts is by pairing them with an algal partner that has a higher thermotolerance (Kinzie et al., 2001; Fautin and Buddemeier, 2004; van Oppen et al., 2009). While this avenue shows promise, it is only functional and applicable if the partnership is successful and can be maintained at higher temperatures. Following our adaptive bleaching trials using Symbiodiniaceae strains that maintained a symbiosis with *C. xamachana* polyps (*Smic, Stri*, *Slin*, *Spil*), we observed that the ability of these strains to resist breakdown under high temperatures can be different *in hospite*. It is important to note that the expulsion of algal cells in our study was also likely facilitated somewhat by lack of feeding and light (though a small percentage). It has been shown that heterotrophic feeding contributes to a host’s ability to resist bleaching in the long-term and the ability to recover at a faster rate (Grottoli et al., 2006; Hughes and Grottoli, 2013). Future experiments focusing on recovery following bleaching should aim to determine the impacts of different feeding regimes on the recovery of *C. xamachana* following time at high temperatures.

Although all of the Symbiodiniaceae strains used for our adaptive bleaching trials belong to the genus *Symbiodinium*, the thermal tolerance of these strains differed. In a study performed on Symbiodiniaceae in culture by Díaz-Almeyda et al. (2017), ITS2 type A3 (*Stri*) *Symbiodinium* was found to be much more sensitive to high temperatures than ITS2 types A1, A2, and A4 (*Smic, Spil*, and *Slin*). In *C. xamachana* polyps, however, *Stri* was found to display a tolerance to higher temperatures (at both 32 and 34°C), with algal cell numbers even showing an increase in the majority of polyps inoculated with this strain at 32°C after 3 days at temperature. In contrast, strain *Slin* typically has a high tolerance to heat (Díaz-Almeyda et al., 2017) but showed poor performance in *C. xamachana* polyps when exposed to high temperature (especially at 34°C). This shift in tolerance once exposed to a different host is indicative of how a native strain (*Stri* was isolated from a *Cassiopea spp.* host) can perform better than a non-native strain (*Slin* was isolated from *Aiptasia pallida*), due to a more favorable host-partner association. Results similar to these are seen in other studies that have observed that the tolerance of an algal strain can differ when in culture versus *in hospite*, and also differ between host-symbiont combinations (Mieog et al., 2009; Díaz-Almeyda et al., 2017; Hoadley et al., 2019). Algal thermotolerance has also been shown to differ between studies (likely due to the host in which a strain is paired with), and work has been done to create a more general ranking of algal tolerance to high temperature (Swain et al., 2017). With regard to partner specificity, Gabay et al. (2019) tested the performance of native Symbiodiniaceae partners against non-native partners in polyps of *Aiptasia pallida.* They introduced aposymbiotic *A. pallida* polyps to a native strain of Symbiodiniaceae that exhibited a lower thermal tolerance concurrently with non-native strains that exhibited a higher thermal tolerance. Following inoculations, it was determined that even at higher temperatures *A. pallida* polyps were more able to readily acquire the thermally sensitive native Symbiodiniaceae strain. These results for *A. pallida* polyps mirror those in our trials with *C. xamachana* polyps; in both cases, pairing a cnidarian host with a native Symbiodiniaceae strain was more successful even at high temperature than introducing a strain that has a higher overall thermotolerance. In general, it appears that a cnidarians hosts’ high specificity for one or a few Symbiodiniaceae types can restrict the acquisition of more thermally tolerant (non-native) algal types even as temperatures increase. Overall, given the evidence of past studies and evidence presented here for *C. xamachana* polyps, a change in symbiont type to a more thermotolerant strain is likely highly restricted. This restriction could mean that reshuffling of algal partners in cnidarian hosts following a bleaching event is not possible for some species, or that reshuffling is limited to native Symbiodiniaceae strains or free-living strains found in an environment in close proximity to a specific cnidarian species.

Previous investigations exploring the adaptive capacity of symbiotic cnidarians have shown that strains of Symbiodiniaceae better adapted to high temperatures can, in fact, recolonize cnidarian hosts following a bleaching event, though these new strains are usually ones naturally found in a species prior to bleaching (Berkelmans and van Oppen, 2006; Jones et al., 2008). Jones et al. (2008) determined that, for example, *Acropora millepora* colonies found on the Great Barrier Reef contained either a Symbiodiniaceae strain sensitive to heat (*Cladocopium spp.*) or a strain that was more thermotolerant (*Durusdinium spp.*). The colonies that harbored *Cladocopium* were able to recover after being bleached and establish a new symbiosis with relatively heat tolerant *Durusdinium* strains (thereby increasing the overall tolerance of the cnidarian-symbiont pairing). However, there was no reshuffling to accommodate a Symbiodiniaceae strain not found originally in the *A. millepora* population (LaJeunesse et al., 2003). A similar finding was reported by Berkelmans and van Oppen (2006). These researchers demonstrated that transplanted *A. millepora* colonies on the Great Barrier Reef were able to increase their thermotolerance by switching from a predominant symbiosis involving *Cladocopium* strain to one with a *Durusdinium* strain. Although this change was beneficial to *A. millepora* colonies, the shift from a sensitive strain to a tolerant strain was due to the restructuring of Symbiodiniaceae strains already found in the tissues of these coral hosts and not the acquisition of a new strain from the environment. The findings from these studies again highlight the small range in which cnidarian hosts can shift their algal cells populations (especially in the long term).

### Long Term Environmental Impacts

The results presented from our adaptive bleaching trials using the cnidarian host *C. xamachana* show that the mechanisms of partner reshuffling are not as flexible as previously thought. We provide evidence that although some *C. xamachana* have the ability to form symbioses with multiple strains of Symbiodiniaceae, including strains that have a higher thermotolerance, high partner specificity with certain strains can impact how well a non-native (more tolerant) strain performs at high temperatures when inside host tissues. As observed at the end of our adaptive bleaching trials, each experimental polyp group ultimately saw expulsion of the majority of algal cells. This suggests that even though some strains maintain a higher thermotolerance outside of the host, this increased thermotolerance does not translate well when the symbiont strain is paired with a non-native partner (or a host that it does not create an ideal partnership with). Future work should be aimed not only at testing rate of loss of algal cells, but also testing the physiological performance of these strains in *C. xamachana* hosts at high temperatures to create a clearer picture of what is occurring. Overall, these findings indicate that it may not be possible to increase long-term resistance of a cnidarian host by just providing it with a more thermotolerant algal strain. In hosts that display more flexibility during symbiont uptake, however, acquiring new algal partners following a bleaching event could be a promising tactic to help these threatened symbiotic cnidarians better adapt, if only briefly, to a changing climate.

## Data Availability Statement

All datasets generated for this study are included in the article/Supplementary Material.

## Author Contributions

CN designed the study, performed the experiments, analyzed the data and wrote the manuscript. TF, MM, and CS provided expert feedback and edits to the manuscript.

## Conflict of Interest

The authors declare that the research was conducted in the absence of any commercial or financial relationships that could be construed as a potential conflict of interest.
