# Supplementary material for: Adaptation to Bleaching: Are Thermotolerant Symbiodiniaceae Strains More Successful Than Other Strains Under Elevated Temperatures in a Model Symbiotic Cnidarian?
Source: Front Microbiol. 2020 May 5;11:822. doi: 10.3389/fmicb.2020.00822 (PMC7214872; doi:10.3389/fmicb.2020.00822)
Supplement: Supplementary file 1 [file Data_Sheet_1.pdf]

Supplemental Table S1. Algal cell population size in polyps inoculated with strain *Stri* at each time point; kept at 32°C (red= increase)

Supplemental Table S1.

	Day 1	Day 3	Day 9	Day 14
Polyp 1	1927	3018	232	98
Polyp 2	2543	3020	405	35
Polyp 3	3118	4395	314	78
Polyp 4	13297	12985	3543	224
Polyp 5	6940	2976	44	13
Polyp 6	3712	4185	63	35
Polyp 7	1557	1652	487	467
Polyp 8	1854	1877	481	24
Polyp 9	1689	1725	434	40
Polyp 10	1694	1378	197	69

Supplemental Table S2. Algal cell population size in polyps inoculated with strain *Smic* at each time point; kept at 32°C (red= increase)

Supplemental Table S2.

	Day 1	Day 3	Day 9	Day 14
Polyp 1	8603	6646	2246	639
Polyp 2	8748	3947	176	8
Polyp 3	12060	8710	571	13
Polyp 4	2900	3182	1974	428
Polyp 5	3211	4009	1494	1071
Polyp 6	4017	2991	156	21
Polyp 7	4836	1762	52	35
Polyp 8	12987	10023	415	37
Polyp 9	6919	4326	177	8
Polyp 10	2428	2809	202	10

Supplemental Table S3. Algal cell population size in polyps inoculated with strain *Slin* at each time point; kept at 32°C (purple= strobilation)

Supplemental Table S3.

	Day 1	Day 3	Day 9	Day 14
Polyp 1	6482	720	52	6
Polyp 2	8193	5240	1161	591
Polyp 3	9625	7075	5509	3540
Polyp 4	10722	9761	491	19
Polyp 5	5292	556	204	198
Polyp 6	6095	147	18	4
Polyp 7	5733	3863	714	150
Polyp 8	3834	67	5	0
Polyp 9	3229	2657	661	178
Polyp 10	10477	5806	713	0

Supplemental Table S4. Algal cell population size in polyps inoculated with strain *Spil* at each time point; kept at 32°C (red= increase, purple= strobilation)

Supplemental Table S4.

	Day 1	Day 3	Day 9	Day 14
Polyp 1	4897	4084	4009	874
Polyp 2	3048	870	133	53
Polyp 3	6179	5255	625	86
Polyp 4	5494	6741	412	41
Polyp 5	8195	8098	2088	1548
Polyp 6	9700	9654	460	21
Polyp 7	3810	1758	16	16
Polyp 8	1050	758	346	120
Polyp 9	1255	1099	533	109
Polyp 10	1075	967	256	36

Supplemental Table S5. Algal cell population size in wild type polyps at each time point; kept at 32°C (red= increase)

Supplemental Table S5.

	Day 1	Day 3	Day 9	Day 14
Polyp 1	2036	2500	2313	756
Polyp 2	1962	2200	699	7
Polyp 3	2089	3068	700	157
Polyp 4	11966	8079	6281	5867
Polyp 5	15946	5454	285	4
Polyp 6	1844	1794	25	3
Polyp 7	20121	17493	4217	4065
Polyp 8	2629	2577	1571	1059
Polyp 9	1992	1417	135	45
Polyp 10	2750	2298	1080	480

Supplemental Table S6.

	Day 1	Day 3	Day 9	Day 14
Polyp 1	2143	2141	148	8
Polyp 2	7673	7669	1074	43
Polyp 3	25247	20208	867	79
Polyp 4	16883	12234	3997	96
Polyp 5	8081	4116	(Dead)	(Dead)
Polyp 6	3752	1365	223	6
Polyp 7	3783	2691	65	1
Polyp 8	2435	1547	548	28
Polyp 9	2179	1749	605	19
Polyp 10	2509	2041	484	63

Supplemental Table S7.

	Day 1	Day 3	Day 9	Day 14
Polyp 1	7752	2638	576	52
Polyp 2	2588	762	386	37
Polyp 3	6991	3633	573	118
Polyp 4	7235	3661	529	88
Polyp 5	9154	8894	2680	161
Polyp 6	4388	3535	320	246
Polyp 7	7436	3527	430	10
Polyp 8	11992	4388	509	48
Polyp 9	5477	1531	101	4
Polyp 10	1415	1348	149	3

Supplemental Table S8.

	Day 1	Day 3	Day 9	Day 14
Polyp 1	8520	201	26	1
Polyp 2	3075	1305	111	65
Polyp 3	4284	149	49	2
Polyp 4	8639	2908	176	15
Polyp 5	7326	1901	13	5
Polyp 6	4072	847	51	2
Polyp 7	6857	573	43	1
Polyp 8	3337	881	118	0
Polyp 9	6903	3830	306	(Dead)
Polyp 10	8685	2626	268	0

Supplemental Table S9.

	Day 1	Day 3	Day 9	Day 14
Polyp 1	17966	4084	1560	456
Polyp 2	8423	870	741	323
Polyp 3	8022	5255	1972	169
Polyp 4	6902	6732	88	90
Polyp 5	7851	4737	540	13
Polyp 6	6700	3609	1090	34
Polyp 7	2448	2180	239	96
Polyp 8	4619	2233	253	8
Polyp 9	1262	1223	25	8
Polyp 10	6344	5564	1190	56

Supplemental Table S10.

	Day 1	Day 3	Day 9	Day 14
Polyp 1	2070	1343	126	5
Polyp 2	1953	1583	261	182
Polyp 3	19295	(Dead)	(Dead)	(Dead)
Polyp 4	10569	6756	37	0
Polyp 5	2078	1132	460	75
Polyp 6	10055	4787	54	0
Polyp 7	1925	1051	549	107
Polyp 8	1573	1236	242	14
Polyp 9	15569	4228	38	5
Polyp 10	1275	1331	101	0

Supplemental Table S6. Algal cell population size in polyps inoculated with strain *Stri* at each time point; kept at 34°C

Supplemental Table S7. Algal cell population size in polyps inoculated with strain *Smic* at each time point; kept at 34°C

Supplemental Table S8. Algal cell population size in polyps inoculated with strain *Slin* at each time point; kept at 34°C

Supplemental Table S9. Algal cell population size in polyps inoculated with strain *Spil* at each time point; kept at 34°C

Supplemental Table S10. Algal cell population size in wild type polyps at each time point; kept at 34°C (red= increase)

Supplemental Table 11.

Least square means comparison (LSMEAN) between groups

**Groups being compared**	**Estimate at 32°C**	**Pr(>|t|) at 32°C**	**Estimate at 34°C**	**Pr(>|t|) at 34°C**
Stri- *Slin*	0.118612	0.7997	0.98028	**0.0161**
Stri – *Smic*	-0.268122	0.5669	-0.30227	0.4462
Stri -*Spil*	-0.176986	0.7052	-0.43890	0.2701
Stri - Wild type	-0.565850	0.2294	0.86194	**0.0332**
Slin – *Smic*	-0.386734	0.4096	-1.28255	**0.0020**
Slin - *Spil*	-0.295598	0.5280	-1.41918	**0.0007**
Slin - Wild type	-0.684463	0.1474	-0.11835	0.7649
Smic - *Spil*	0.091136	0.8454	-0.13664	0.7299
Smic - Wild type	-0.297728	0.5250	1.16420	**0.0047**
Spil - Wild type	-0.388864	0.4071	1.30084	**0.0018**

Estimate refers to how strains are performing at temperature in comparison to one another. A negative value indicates that the second strain listed is outperforming the first strain. P<0.05 indicates a statistically significant difference between groups.
